# Comparison of a teratogenic transcriptome-based predictive test based on human embryonic versus inducible pluripotent stem cells

**DOI:** 10.1186/s13287-016-0449-2

**Published:** 2016-12-30

**Authors:** Vaibhav Shinde, Sureshkumar Perumal Srinivasan, Margit Henry, Tamara Rotshteyn, Jürgen Hescheler, Jörg Rahnenführer, Marianna Grinberg, Johannes Meisig, Nils Blüthgen, Tanja Waldmann, Marcel Leist, Jan Georg Hengstler, Agapios Sachinidis

**Affiliations:** 1 Institute of Neurophysiology and Center for Molecular Medicine Cologne (CMMC), University of Cologne (UKK), Robert-Koch-Str. 39, 50931 Cologne, Germany; 2 Department of Statistics, Technical University of Dortmund University, 44227 Dortmund, Germany; 3 Integrative Research Institute for the Life Sciences, Institute for Theoretical Biology, Humboldt University, 10115 Berlin, Germany; 4 Doerenkamp-Zbinden Chair for In Vitro Toxicology and Biomedicine, University of Konstanz, Box: M657, 78457 Konstanz, Germany; 5 Leibniz Research Centre for Working Environment and Human Factors at the Technical University of Dortmund (IfADo), 44139 Dortmund, Germany

**Keywords:** Embryonic stem cells, Induced pluripotent stem cells, Differentiation, Genomics, Cytotoxic agents, Embryoid bodies

## Abstract

**Background:**

Human embryonic stem cells (hESCs) partially recapitulate early embryonic three germ layer development, allowing testing of potential teratogenic hazards. Because use of hESCs is ethically debated, we investigated the potential for human induced pluripotent stem cells (hiPSCs) to replace hESCs in such tests.

**Methods:**

Three cell lines, comprising hiPSCs (foreskin and IMR90) and hESCs (H9) were differentiated for 14 days. Their transcriptome profiles were obtained on day 0 and day 14 and analyzed by comprehensive bioinformatics tools.

**Results:**

The transcriptomes on day 14 showed that more than 70% of the “developmental genes” (regulated genes with > 2-fold change on day 14 compared to day 0) exhibited variability among cell lines. The developmental genes belonging to all three cell lines captured biological processes and KEGG pathways related to all three germ layer embryonic development. In addition, transcriptome profiles were obtained after 14 days of exposure to teratogenic valproic acid (VPA) during differentiation. Although the differentially regulated genes between treated and untreated samples showed more than 90% variability among cell lines, VPA clearly antagonized the expression of developmental genes in all cell lines: suppressing upregulated developmental genes, while inducing downregulated ones. To quantify VPA-disturbed development based on developmental genes, we estimated the “developmental potency” (*D*_*p*_) and “developmental index” (*D*_*i*_).

**Conclusions:**

Despite differences in genes deregulated by VPA, uniform *D*_*i*_ values were obtained for all three cell lines. Given that the *D*_*i*_ values for VPA were similar for hESCs and hiPSCs, *D*_*i*_ can be used for robust hazard identification, irrespective of whether hESCs or hiPSCs are used in the test systems.

**Electronic supplementary material:**

The online version of this article (doi:10.1186/s13287-016-0449-2) contains supplementary material, which is available to authorized users.

## Background

Drug-induced embryotoxicity, manifested as teratogenicity, is a major safety issue. At present, various in vivo and in vitro assays are used for testing for adverse teratogenic effects of potential drug candidates. However, the present transitional teratogenicity assessment methods are limited because: (1) interspecies differences in both in vitro and in vivo animal-based test systems do not optimally predict human relevant teratogenic drug candidates; (2) traditional methodologies involve extensive animal studies, making tests costly and time-consuming; and (3) traditional approaches are not efficient given that they only allow testing of a limited number of compounds at a time, even though the number of the drug candidates increases markedly each year (http://cen.acs.org/articles/94/i5/Year-New-Drugs.html). Such limitations have resulted in several drugs being withdrawn from the market because of toxic effects to humans [1]. To overcome these limitations novel in vitro testing systems are urgently needed [2–7]. Recently, tremendous efforts have been made to develop in vitro test systems for identifying teratogenic effects of drug candidates based on human embryonic stem cells (hESCs) and human induced pluripotent stem cells (hiPSCs), as reviewed in [8–10]. Both in vitro hESC-based systems developed by the University of Konstanz (UKN) and Universitätsklinikum Köln (UKK) recapitulate the critical phases of embryonic development, during which cells can be exposed to various test compounds [11]. These systems have already been applied in numerous studies to identify and characterize developmental toxicants [12–15].

More recently, the UKN and UKK test systems have been upgraded to so-called -omics prediction test systems (STOP-Tox), allowing quantification of the developmental toxicity of a compound, based on microarray gene expression data [10]. In the UKK test system, which partially recapitulates early embryonic development at transcriptomics level, H9 hESCs were randomly differentiated for 14 days to three germ layers and their derivatives [13–15]. The differential regulated genes on day 14 of differentiation compared with undifferentiated hESCs (day 0) were identified using genome-wide microarrays and were designated as “developmental” probe sets or “developmental” genes. Moreover, the influence of six mercurials and six histone deacetylase inhibitors on these developmental genes was quantified using two basic indices, “developmental potency” (*D*_*p*_) and “developmental index” (*D*_*i*_). Both *D*_*i*_ and D_p_ quantitatively predict and discriminate the toxicity effects of various chemicals on embryonic development. This recently developed STOP-Tox_UKK_ test is based on hESCs [10]. However, there is an ongoing ethical debate over the use of hESCs for embryotoxicity testing [16]. The discovery of hiPSCs [17] provides an alternative to hESCs for toxicity testing. In this context, very few studies are available applying hiPSCs as a model for developmental neurotoxicity (for review see [18, 19]). Although hiPSCs are most similar to hESCs, small differences still exist in their epigenetic landscape, transcribed genes, and differentiation potential [20]. In the present study, we investigated whether hESCs can be replaced by hiPSCs to develop a sensitive developmental test system. Here, we systematically compare the developmental toxicity potency of valproic acid (VPA) on two hiPSC-based cell lines (foreskin and IMR90) along with H9, using transcriptomics and comparative bioinformatics.

## Methods

### Materials

The H9 hESCs (as WA09 line), foreskin hiPSCs (clone 4) and IMR90 hiPSCs (clone 4) were obtained from WiCell (Madison, WI, USA). H9 hESCs were cultured on irradiated mouse embryonic fibroblasts in a culture medium, as described in [15]. BD Matrigel matrix (354277) and BD Matrigel growth factor reduced (354230) used for culturing were from BD Biosciences (San Jose, CA, USA). All cell culture reagents were from Gibco/Invitrogen (Darmstadt, Germany), unless otherwise specified. VPA (P4543) and Pluronic F-127 (P2443) were obtained from Sigma-Aldrich (Steinheim, Germany).

### Random differentiation of stem cells to germ layer cell types and their derivatives

To remove the mouse embryonic fibroblasts, the H9 hESCs were transferred from the maintenance culture onto hESC-qualified matrix (BD Biosciences) -coated 60-mm tissue culture plates (Nunc, Langenselbold, Germany) in TESR1 medium (Stem Cell Technologies, Vancouver, BC, Canada). The hiPSCs (foreskin and IMR-90) were maintained on 60-mm tissue culture plates coated with BD Matrigel growth factor reduced in TESR1 medium. Cells were maintained on these plates for 5 days prior to differentiation. The random differentiation of hESCs was performed using the embryoid bodies protocol, as described previously [15]. Briefly, the clumps were obtained by cutting and scraping the cells with passage scrapers (StemPro EZPassageTM Disposable; Invitrogen, Carlsbad, CA, USA). On day 0, 100 clumps were seeded in a conical well, coated with Pluronic F-127 (5%) in 100 μl of random differentiation medium (Dulbecco’s modified Eagle’s medium (DMEM)-F12 medium with 20% KO serum replacement, 1% non-essential amino acids, penicillin (100 units/ml), streptomycin (100 μg/ml), 0.1 mM β-mercaptoethanol) containing 1 mM VPA or vehicle, and incubated for 4 days at 37 °C and 5% CO_2_. The embryoid bodies were collected on day 4 and transferred onto 100-mm bacteriological plates in 15 ml of random differentiation medium containing 1 mM VPA or vehicle. The medium was replenished every alternate day, until day 14.

### Microarray experimental details

Cell RNA isolation was performed, as previously reported [14, 21]. Briefly, total RNA was isolated using TRIzol and chloroform (Sigma-Aldrich) and purified with miRNeasy mini kit (Qiagen, Hilden, Germany). All quantification and quality measurements were performed using a NanoDrop spectrophotometer (ND-1000; Thermo Fisher Scientific, Langenselbold, Germany). For microarray labelling, 100 ng total RNA was taken as a starting material, and after amplification, 12.5 μg-amplified RNA was hybridized on Affymetrix Human Genome U133 Plus 2.0 arrays (Affymetrix, Santa Clara, CA, USA). For washing and staining, Affymetrix HWS kit and Genechip Fluidics Station 450 were used, according to the manufacturer’s instructions. After staining, arrays were scanned with Affymetrix GeneChip Scanner 3000 7G and Affymetrix GCOS software was used for quality control analysis.

### Statistical data and functional annotation analysis

Microarrays statistical data analysis and visualization were carried out by uploading. CEL files in Partek Genomics Suite (PGS) version 6.6 (Partek, St. Louis, MO, USA). The probe sets intensity values were obtained after RMA background correction, quantile normalization, log2 transformation, and median polished probe sets summarization. The normalized probe sets were used for principal component analysis (PCA), while a one-way ANOVA model was used to generate the differentially regulated transcripts, with at least a twofold change using the Benjamini and Hochberg false discovery rate (FDR) correction (*p* ≤ 0.05). The signals of differentially regulated probe sets were normalized using their Z scores and were clustered using unsupervised hierarchical cluster analysis. The Database for Annotation, Visualization and Integrated Discovery (DAVID) was used for gene ontology categories (GOs) and Kyoto Encyclopedia of Genes and Genomes (KEGG) pathway analysis of differentially expressed transcripts [22, 23].

*D*_*p*_ and *D*_*i*_ were calculated as per the formula

${\displaystyle {D}_p}=\frac{O}{D}$ and ${\displaystyle {D}_i}=\frac{O\times A}{T\times D}$ (for details of the terms please refer Fig. 3).

## Results

### Data structure of developmental and valproic acid-deregulated genes in differentiating stem cells

**Fig. 1:**
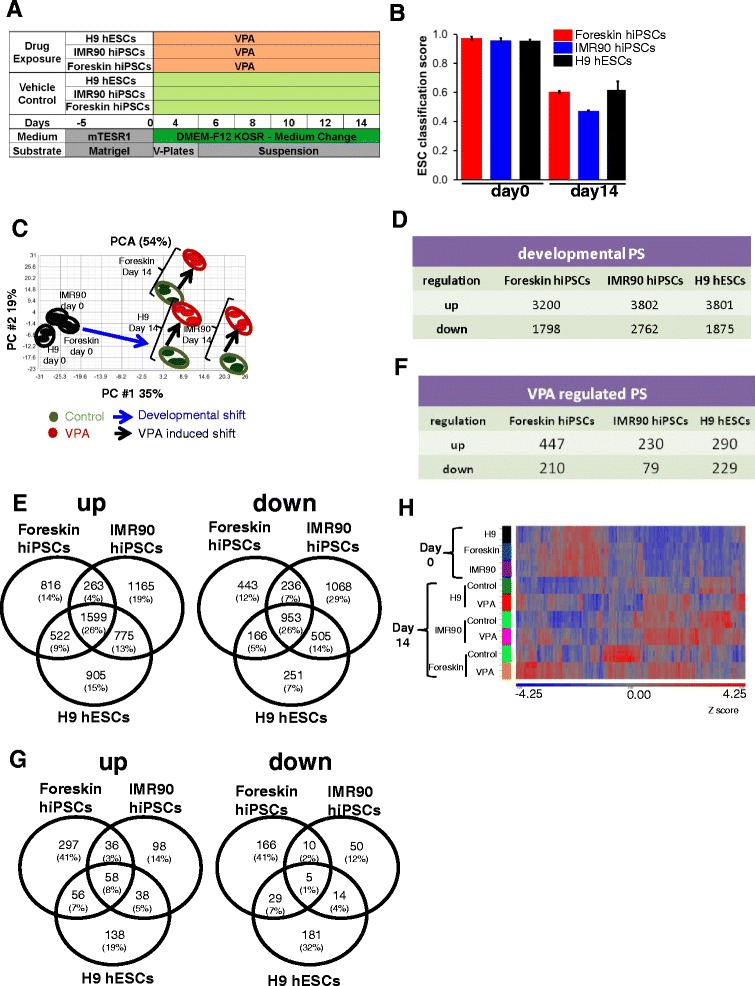
Global analysis of the valproic acid (VPA)-induced differentially expressed genes in human embryonic stem cells (hESCs) and human induced pluripotent stems cells (hiPSCs). **a** hESCs and hiPSCs were differentiated towards all three germ layers and their derivatives for 14 days in the presence and absence of VPA. Samples from three biological replicates were collected on day 0 and day 14 for the microarray studies. **b** CellNet analysis of the.CEL files shows the ESC classification score, which represents the pluripotency status of hESCs and hiPSCs on day 0 and differentiated cells at day 14. **c** Two-dimensional principle component analysis (2D-PCA) of differentially expressed genes (hESCs or hiPSCs at day 0 vs 14 days of differentiation, absolute fold change ≥ 2, *p* < 0.05; 14-day differentiated hESCs or hiPSCs in the presence and absence of VPA, with absolute fold change ≥ 2, *p* < 0.05). The PCA illustrates a significant variance in the gene expression level in PC1 from day 0 to day 14 in the absence of VPA, whereas PC2 represents variance in the expression level of genes induced by VPA. **d** “Developmental” probe sets are defined as differentially expressed probe sets on day 14 of differentiation, compared with undifferentiated H9 hESCs, values are for foreskin hiPSCs and the IMR90 hiPSCs on day 0 (absolute fold change ≥ 2, FDR corrected *p* value < 0.05). **e** Venn diagram of the developmental PS, showing up- and downregulated genes. **f**, **g** Numbers and Venn diagrams of the differentially expressed probe sets (absolute fold change ≥ 2, FDR-corrected *p* value < 0.05) after exposure to VPA for 14 days, compared with 14-day differentiated H9 hESCs, foreskin hiPSCs and IMR90 hiPSCs. **h** Hierarchical cluster analysis of significantly deregulated transcripts (absolute fold change ≥ 2, *p* < 0.05) in 14-day untreated versus VPA-treated cells (Partek Genomics Suite). The results are represented as a heatmap, with gene expression level of the probe set given by *blue*: low; and *red*: high

Both hESCs and hiPSCs were differentiated for 14 days. Gene expression profiles were analyzed for hESCs and hiPSCs on day 0 and day 14 (Fig. 1a). To compare the differentiation potential of hESCs with hiPSCs and to quantify their resemblance with specific human cell types, we performed a gene regulatory network analysis using CellNet [24]. CellNet showed embryonic stem cell (ESC) scores higher than 0.95 for hESCs and hiPSCs on day 0, indicating relatively similar transcriptome profile of hESC and both hiPSC cell lines with standard hESCs. Differentiation over 14 days resulted in a significant decrease in the ESC score, indicating variable differentiation of hESCs and hiPSCs. IMR90 hiPSCs had lower ESC scores than foreskin hiPSCs and H9 hESCs (Fig. 1b). Although the increase in tissue classification scores was relatively small for all three cells lines, there were a few striking differences observed among them (Additional file 1: Figure S1). On day 0 and day 14 of differentiation, foreskin and IMR90 hiPSCs showed higher scores for fibroblasts than hESCs. Similarly, their scores were higher for lung on day 14. IMR90 had highest scores for skin, heart and kidney on day 14 of differentiation (Additional file 1: Figure S1). During differentiation, cells were exposed to 1 mM VPA from day 0 to day 14 and gene expression profiles from day 0 and 14 were compared with time-matched controls (Fig. 1a). To obtain an overview of this genome-wide data, PCA plots were prepared. Differentiation over 14 days resulted in relatively large spread within the PCA plot, although all three cell types shifted in a similar direction under treatment (Additional file 1: Figure S2). PCA plots based on differentially regulated genes on day 14 compared with day 0 (Additional file 2: Table S1) and differential genes induced by VPA on day 14 (Additional file 2: Table S2) were prepared. This PCA plot shows only small differences between hESCs and hiPSCs on day 0 (Fig. 1c). Our analysis illustrates a relatively large shift among the controls for all three cell lines between day 0 and day 14, occurring along the first principle component axis. In contrast, VPA-induced effects led to a shift predominantly along the second principle component axis (Fig. 1c). The differentially regulated genes on day 14 with respect to day 0 (having an absolute fold change ≥ 2 and FDR-corrected *p* value < 0.05) in H9 hESCs, IMR90 and foreskin hiPSCs are further referred as “developmental genes” (Fig. 1d). The number of developmental genes was much higher than the genes deregulated by VPA on day 14 (Fig. 1f). The overlap of developmental genes among the three cell lines shows that 26% are common up- or downregulated genes that means variability amongst them is > 70% (Fig. 1e). In contrast, overlap of VPA-deregulated genes captured only 8% of up- and 1% of downregulated genes among hESCs and hiPSCs that means variability amongst them is > 90% (Fig. 1g). Cluster analysis based on Z scores for VPA-deregulated genes (absolute fold change ≥ 2, FDR-corrected *p* value < 0.05) led to distinct clusters for day 0 and day 14, irrespective of cell line (Fig. 1h). VPA-exposed samples did not lead to independent branches, but rather clustered close to their respective controls (Fig. 1h).

### Characterization of developmental genes of stem cells

**Fig. 2:**
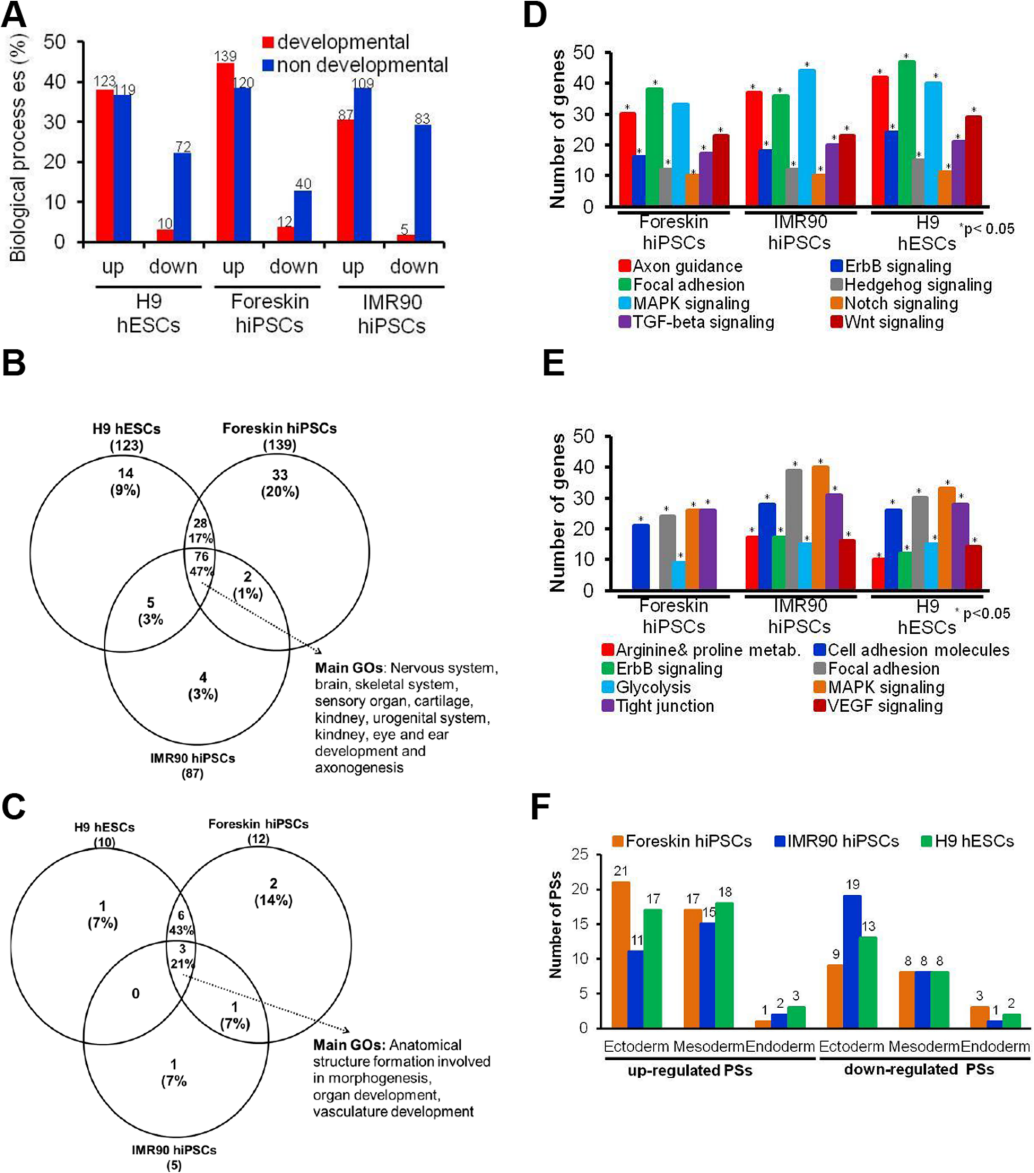
Characterization of differentially regulated probe sets at day 14 of differentiation in the human embryonic stem cells (hESCs) and human induced pluripotent stem cells (hiPSCs). **a** The differentially expressed (absolute fold change ≥ 2, false discovery rate (FDR)-corrected *p* value < 0.05) probe sets at day 14, called developmental probe sets were further characterized using the online tool ‘DAVID’. The gene ontology categories (GOs) belonging to biological processes (BPs) overrepresented among the up- and downregulated probe sets (*p* < 0.05) were further subcategorized into two classes: “developmental” and “non-developmental” GOs. The numbers of the overrepresented GOs for up- and downregulated genes in hESCs and hiPSCs are shown on the top of each bar. **b**, **c** Venn diagrams indicating the intersections for up- or downregulated developmental GOs, among hESCs and hiPSCs, respectively. **d**, **e** KEGG pathways associated with the up- or downregulated developmental probe sets, respectively. Numbers indicate the total number of probe sets (*FDR-corrected; *p* value < 0.05). **f** Overlap analysis of the well-annotated three germ layer-specific genes obtained from Partek Genomics Suite, showing overlap among the developmental genes. The total number of the deregulated probe sets is indicated on the top of each column

The biological processes significantly influenced by developmental genes were assessed for overrepresented GOs (Additional file 2: Tables S3 and S4). Overrepresented GOs were subdivided into developmental (associated with embryonic development) or non-developmental GOs (associated with cellular homeostasis). Downregulated developmental genes contributed to less than 4% developmental and less than 25% non-developmental GOs, whereas upregulated developmental genes contributed to more than 30% developmental and non-developmental GOs in H9 hESCs versus foreskin and IMR90 hiPSCs (Fig. 2a). These results demonstrate that a similarly high number of developmental and non-developmental GOs are covered by upregulated developmental probe sets in all three cell lines. In contrast, only a few developmental GOs have been identified among the downregulated PS, although a relatively high number of non-developmental genes occur in all three differentiated cell lines. These results show that more upregulated developmental GOs are necessary to drive all three cell lines to more specialized somatic cell types than downregulated developmental GOs, which are necessary only for regulation of the pluripotent stage. Consequently, because general non-developmental processes associated with cellular homeostasis (e.g., metabolism, cell proliferation) occur in all cell types, a high number of downregulated non-developmental GOs would also be expected. Overlap analysis of GOs captured by upregulated developmental genes shows almost 47% overlap between hESCs and hiPSCs (Fig. 2b). For downregulated developmental genes, only 21% of overrepresented GOs overlap all three cell lines (Fig. 2c). Overall, 64% up- or downregulated GOs were in the overlap region between differentiated H9 ESCs and foreskin hiPSCs; while only 50% of up- and 21% of downregulated genes overlap between H9 hESCs and IMR90 hiPSCs; and 48% of up-, and 28% of downregulated GOs overlap between foreskin and IMR90 hiPSCs (Fig. 2b, c). The higher overlap observed for GOs (Fig. 2b, c) compared to probe sets (Fig. 1e, g) shows that although different genes are involved in hESCs and hiPSCs, they nevertheless fall into similar GOs. For upregulated developmental genes, significantly overrepresented KEGG pathways include focal adhesion, Erb signalling, Wnt signalling, TGF-β signalling, and the Hedgehog pathway in H9 hESCs, as well as in both hiPSCs (Fig. 2d). These pathways are known to be involved in embryonic development. Significantly overrepresented KEGG pathways for downregulated developmental genes include MAPK signalling, tight junction, glycolysis, cell adhesion molecules, and focal adhesion in all three cell lines. Erb signalling, VEGF signalling, arginine and proline metabolism were overrepresented only in H9 hESCs and the IMR90 hiPSCs (Fig. 2e). Moreover, germ layer-specific genes among the developmental genes were analysed using Partek Genomics Suite (Additional file 2: Table S5). High numbers of developmental genes (up- and downregulated) of differentiated hESCs, IMR90 and foreskin hiPSCs were ectoderm- and mesoderm-specific, as opposed to endoderm-specific (Fig. 2f). Common ectodermal upregulated and downregulated genes among all three cell lines were *TFAP2A, ATP7A, PAX6, LEF1, DCT, BCL2, POU3F2, APC, COL5A2 and BTD, PDGFA, FRAS1, PPL, NF2,* respectively. Common upregulated and downregulated mesodermal genes were *DLL3, EXT1, FOXC1, LEF1, LHX2, SMAD3, BMP4, SNAI2, SMAD1, SLIT2* and *NF2, FOXH1, MATK, HCK, TCF7L1*, respectively. The only common upregulated endodermal gene was *EXT1*. These results indicated that all three cell lines mainly captured ectodermal and mesodermal genes within 14 days of differentiation, with several of these genes common among all cell lines.

### Comparison of gene expression in undifferentiated stem cells (day 0)

To compare the pluripotency state of the two hiPSCs with the pluripotency state of hESCs, a comparison of their transcriptomes at day 0 was performed, and differentially regulated genes (absolute fold change ≥ 2, FDR-corrected *p* value < 0.05) were identified (Additional file 2: Table S6). Among the key pluripotency related genes (*POU5F1, NANOG, SOX2,* and *KLF4*), only the expression level of *KLF4* was found higher in foreskin hiPSCs, as compared to H9 hESCs and IMR90 hiPSCs undifferentiated cells, whereas the expression levels of *POU5F1, NANOG, SOX2* was very similar in all three cell lines. There was no significant difference observed for *KLF4* between undifferentiated H9 hESCs and IMR90 hiPSCs. Moreover, five mostly upregulated genes, *DDX3Y, EIF1AY, USP9Y* and *RPS4Y1* (located in the Y chromosome) were highly upregulated in foreskin hiPSCs, compared to H9 ESCs or IMR90 hiPSCs (>60-fold change, *p* value < 7.4E-20). *XIST* (located in the X-chromosome) was found to be markedly downregulated in foreskin hiPSCs, compared to IMR90 hiPSCs and the hESCs. These findings reflect the fact that the foreskin hiPSCs are from a male (karyotype of XY), while both H9 hESCs and IMR90 hiPSCs are from females (XX karyotype).

### Characterization of valproic acid-deregulated genes in stem cells

**Fig. 3:**
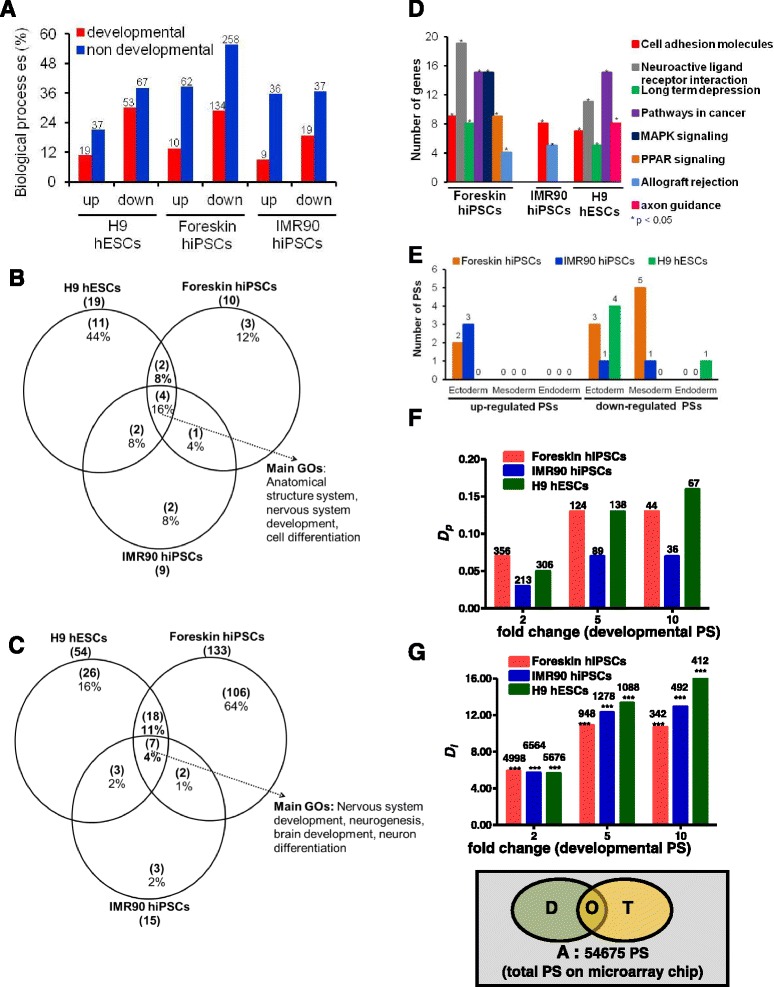
Characterization of valproic acid (VPA)-deregulated probe sets on day 14 of differentiation in human embryonic stem cells (hESCs) and human induced pluripotent stems cells (hiPSCs) using gene ontology categories (GOs) and KEGG pathway analysis, as well as D_p_ and D_i_ indice values. **a** The differentially regulated probe sets on day 14 by VPA were compared with non-treated 14-day differentiated cells (absolute fold change ≥ 2, false discovery rate-corrected *p* value < 0.05). The number of up- and downregulated genes overrepresented in GOs in hESCs and hiPSCs are shown on top of the bars. **b**, **c** Venn diagrams obtained for up- or downregulated developmental GOs, for hESCs and hiPSCs, respectively. **d** KEGG pathways of the VPA regulated up- or downregulated developmental probe sets, respectively. **e** Overlap analysis of well-annotated three germ layer-specific genes obtained from Partek Genomics Suite, showing the overlap among VPA-deregulated “developmental” genes. **f** Values for the *D*_*p*_ index calculated using the ratio O/D, the *D*_*i*_ **g** calculated using the ratio (O x A)/(T x D), and the significance of overlap calculated using Fisher's exact test (^***^ *p* < 0.001). In this figure, *A* represents the total probe sets available on the microarray chip; *O* represents VPA deregulated developmental probe sets; *T* represents the VPA deregulated probe sets; *D* represent “developmental” probe sets deregulated on day 14, compared with day 0. The total number of the deregulated probe sets in (**f**) and (**g**) is indicated on the top of the columns

The influence of VPA on both hiPSC lines and H9 hESCs was small compared with effects related to up- or downregulation of developmental genes during the 14-day differentiation period, as evident from the PCA (Fig. 1c). The number of VPA-deregulated probe sets (Fig. 1f; Additional file 2: Table S2) was relatively low compared to the developmental probe sets (Fig. 1d; Additional file 2: Table S1). The biological processes significantly influenced by VPA also were analysed for overrepresented GOs (Additional file 2: Tables S7 and S8). Overrepresented GOs were further subdivided into developmental or non-developmental GOs. Using this subdivision, we observed that the VPA-induced downregulated genes captured more developmental GOs, compared to VPA-induced upregulated genes in all three cell lines (Fig. 3a). Overlap analysis of GO groups overrepresented in VPA-deregulated genes for all three cell lines showed only 16% overlap for up- and 4% for downregulated probe sets (Fig. 3b, c). The common up- or downregulated GOs (see Additional file 2: Tables S7 or S8, respectively) observed in three cell lines included “anatomical structure”, and “nervous system development” versus “neurogenesis”, “brain development”, and “neuron differentiation”, respectively. KEGG pathway analysis identified “cell adhesion molecules” as an overrepresented motif in all three cell lines (Fig. 3d). In particular, the KEGG pathways regulated by VPA in differentiated hESCs were more similar to the differentiated foreskin hiPSCs than to IMR90 hiPSCs (Fig. 3d). Several common germ layer-specific genes were identified within the developmental genes (Fig. 2f). An Venn diagram of the VPA-deregulated germ layer-specific developmental genes (Fig. 3e) shows that only a few common ectoderm genes were downregulated in all three cell lines, although a few mesodermal genes were downregulated in both hiPSC lines (Fig. 3e; Additional file 2: Table S5). Our results suggest that most of the specific germ-layer formation genes are not deregulated by VPA. Apparently, VPA disturbs the expression of developmental genes that are involved in late, and more specific, differentiation processes related to somatic cells. Overlap analysis of the VPA deregulated genes (Additional file 2: Table S2) with developmental genes (Additional file 2: Table S1) demonstrates that < 15% of the developmental genes were affected by VPA in all three cell lines (Additional file 1: Figure S3). This analysis also revealed that VPA antagonized the expression of developmental genes: suppressing upregulated developmental genes and inducing downregulated developmental genes, irrespective of whether they were hESCs or hiPSCs (Additional file 1: Figure S3).

### *D*_*p*_ and *D*_*i*_ of valproic acid in stem cells

*D*_*p*_ represents the fraction of all developmental genes that are up- or downregulated, using a test compound; while *D*_*i*_ gives the ratio of overrepresentation of developmental genes among all genes deregulated by a test compound. In all three test systems, more than 5% of the developmental genes were deregulated by VPA (*D*_*p*_ > 0.05; Fig. 3f). Moreover, all three cell lines showed more than tenfold overrepresentation of developmental genes among all genes deregulated by VPA (Fig. 3g). Notably, the relative changes of the *D*_*p*_ values (Fig. 3f) remained stable in all three cell lines, independent of fold change values varying from two to ten. In contrast, the relative D_i_ values increased, with increasing fold change (Fig. 3g). Using *D*_*i*_ values makes hazard identification more sensitive, because some test compounds compromise the expression of only a relatively small number of genes, but have a high propensity to specifically deregulate developmental genes. In this case, the test compound generates a low *D*_*p*_, but a high *D*_*i*_ value [10]. We show that all three cell lines allowed us to identify VPA as a teratogenic compound with the same sensitivity (Fig. 3g). However, although the *D*_*p*_ value for IMR90 hiPSCs was lower than foreskin hiPSCs (Fig. 3f), their *D*_*i*_ values are a little higher, having fold changes of five and ten, respectively. Thus, IMR90 hiPSCs may allow teratogenicity testing with a higher sensitivity than foreskin hiPSCs. H9 hESCs had the highest indices values, although their differences with values for foreskin hiPSCs were very small, suggesting that both cell systems are equivalent.

### Effects of valproic acid on developmental and non-developmental genes; correlations between stem cell types

**Fig. 4:**
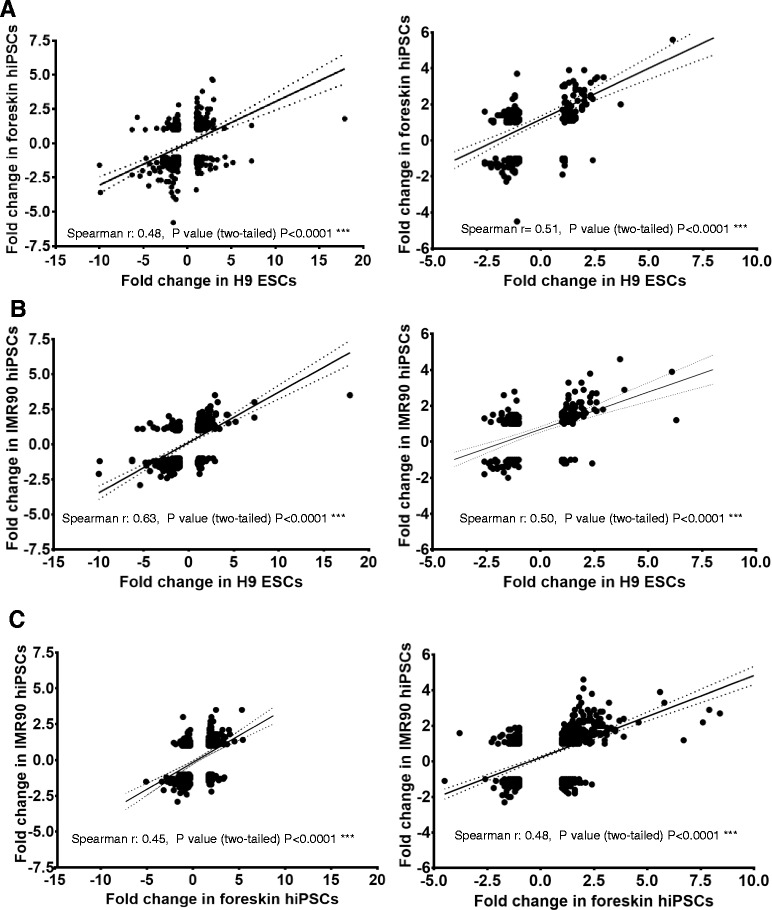
Comparison of deregulated common developmental probe sets having at least a fivefold change in expression on day 14 for human embryonic stem cells (hESCs) and human induced pluripotent stem cells (hiPSCs) affected by valproic acid (VPA).VPA-deregulated fold change values are plotted on x and y axes. **a** Upregulated (*left diagram*) or downregulated (*right diagram*) developmental genes having at least a fivefold expression change, common to 14-day differentiated hESCs (x-axis) versus the 14-day differentiated foreskin hiPSCs. **b** Upregulated (*left diagram*) or downregulated (*right diagram*) developmental” genes having at least a fivefold change in expression, common to 14-day differentiated hESCs (x-axis) and 14-day differentiated IMR90 hiPSCs (y-axis). **c** Upregulated (*left diagram*) or downregulated (*right diagram*) developmental genes having at least a fivefold change in expression common to 14-day differentiated foreskin hiPSCs (x-axis) and 14-day differentiated IMR90 hiPSCs

**Fig. 5:**
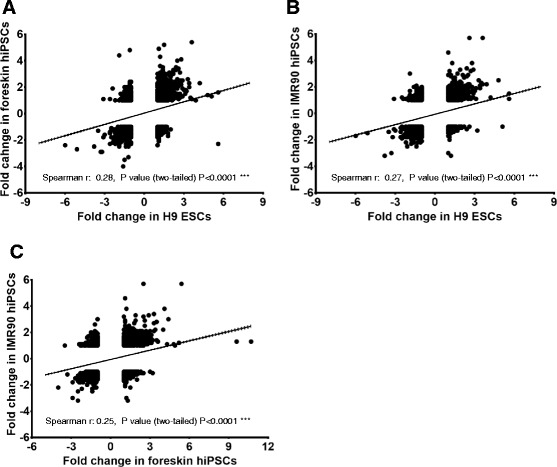
Comparison of common “non-developmental” deregulated genes on day 14 having gene expression levels below twofold (up- and downregulated) in human embryonic stem cells (hESCs) and human induced pluripotent stem cells (hiPSCs) affected by valproic acid (VPA). VPA-deregulated fold change values were plotted on x and y axes. **a** Common “non-developmental” genes in 14 day differentiated hESCs (x-axis) were plotted against the 14-day differentiated foreskin hiPSCs. **b** Common “non-developmental” genes in 14-day differentiated hESCs (x-axis) were plotted against 14-day differentiated IMR90 hiPSCs. **c** Common “non-developmental” genes in 14-day differentiated foreskin hiPSCs (x-axis) were plotted against the 14-day differentiated IMR90 hiPSCs

To determine the influence of VPA on developmental probe sets and their pairwise correlation between the two cell lines, scatter plots were constructed by plotting VPA-deregulated developmental probe sets (fold change values) from one cell line (x-axis) versus another cell line (y-axis) (Fig. 4). According to Spearman’s rank-order criteria, ρ values from 0 to 0.19, 0.20 to 0.39, 0.40 to 0.59, 0.60 to 0.79 and 0.80 to 1.0 show a very weak, weak, moderate, strong, and very strong correlation, respectively. The values obtained from Spearman’s ρ values indicate a moderate correlation between the VPA-deregulated genes in foreskin hiPSCs versus H9 hESCs (Fig. 4a), as well as between foreskin versus IMR90 hiPSCs (Fig. 4c). In contrast, a strong correlation was obtained between VPA deregulated genes in H9 hESCs and IMR90 hiPSCs (Fig. 4b). To assess the influence of VPA on non-developmental PS, and determine any correlations between cell lines, scatter plots were constructed using VPA-deregulated non-developmental probe sets (having an absolute fold change values < 2) (Fig. 5). The ρ values obtained from a Spearman’s rank-order correlation indicate a weak correlation between foreskin hiPSCs versus H9 hESCs (Fig. 5a), IMR90 hiPSCs versus H9 hESCs (Fig. 5b), as well as between foreskin and IMR90 hiPSCs (Fig. 5c). Thus, Spearman’s rank-order analysis indicates that VPA preferably deregulated developmental genes, yielding fold changes greater than five for all three cell lines (i.e., having ρ values indicating moderate to strong correlation). In contrast, non-developmental genes showed a weak correlation, having absolute fold change values below two.

### Key developmental genes influenced by valproic acid in all three cell lines

**Fig. 6:**
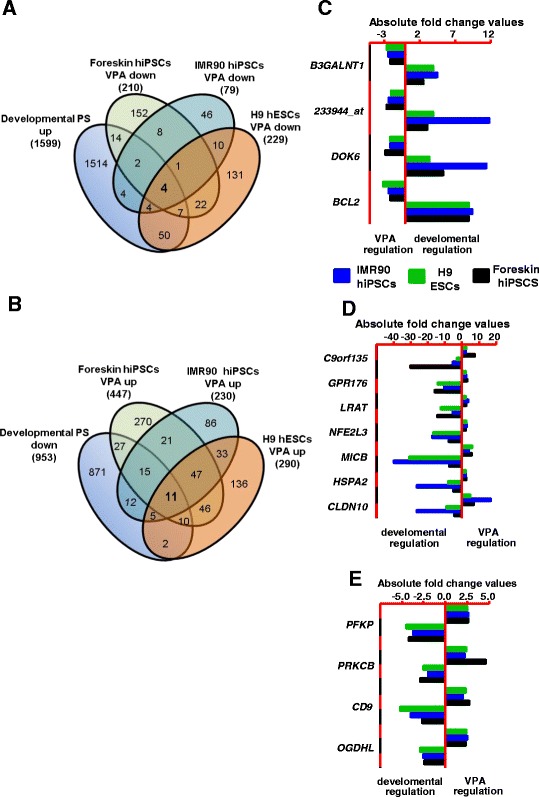
Comparison of the common developmental genes observed at day 14 in all three cell lines in the absence of valproic acid (VPA) with the VPA-deregulated genes at day 14 in each cell line (absolute fold change > 2, false discovery rate-corrected *p* value < 0.05). **a** Overlap of the developmental upregulated genes with the VPA-induced downregulated genes (four genes; see *C* for fold changes). **b** Overlap of developmental downregulated genes with the VPA-induced upregulated genes (11 genes; see *D* and *E* for fold changes). **c** Fold change values for *B3GALNT1*, 233944_at, *DOK6*, and *BCL2* for all three cell lines. **d** Fold change values for *C9orf135*, *GPR176, LRAT, NFE2L3, MICB, HSPA2,* and *CLDN10* for all three cell lines*.* **e** Fold change values for *PFKP, PRKCB, CD9* and *OGDHL* for all three cell lines

To determine VPA-antagonized common developmental genes in hiPSCs and hESCs, an overlap analysis was performed for developmental upregulated or downregulated genes in all three cell lines, for which expression was regulated by VPA in opposing ways (Fig. 6a, b). Four common developmentally upregulated transcripts, i.e., three genes (namely *B3GALNT1*, *DOK6* and *BCL2*) and one unannotated transcript (Affymetrix id: 233944_at), were downregulated by VPA (Fig. 6c). Likewise, 11 common developmentally downregulated transcripts, i.e., ten genes (namely *GPR176, LRAT, NFE2L3, MICB, HSPA2, CLDN10, PFKP, PRKCB, CD9,* and *OGDHL*) and one unannotated transcript (Fig. 6d, e), were upregulated by VPA. These 15 genes have been identified as the top developmental toxicity markers for VPA-induced toxicity in the UKK test system.

## Discussion

Recent evidence suggests that hESCs combined with a transcriptomic approach have the potential to predict human relevant embryotoxicity. In this context, we have developed in vitro methods based on hESCs, using transcriptomics to predict developmental toxicity of different classes of developmental toxicants. Specifically, we tested six histone deacetylase inhibitors and six mercury compounds [11, 12, 15, 25]. Human teratogenic drugs, like thalidomide and VPA, also have been tested using the UKK test system, covering early and late differentiation processes of hESCs. Their altered transcriptomic profiles determined the teratogenic mechanisms for these drugs [13, 14], resolving the in vivo teratogenic effect of these drugs. Furthermore, to quantify levels of developmental toxicity, the indices *D*_*p*_ and *D*_*i*_ were established, using several differentially expressed genes induced by teratogenic compounds, such as thalidomide and VPA [10]. Because of the ethical concerns regarding the use of hESCs, hiPSCs were investigated as an alternative for human relevant in vitro toxicity testing of potential developmental toxicants. Here, we compared transcriptome responses of two different hiPSCs (foreskin and IMR90 hiPSCs) with hESCs, using the UKK test system, and VPA as a developmental toxicant. We quantified the toxicity potential of VPA using both D_p_ and D_i_. According to the UKK test system, cells from all three cell lines were differentiated for 14 days in the presence and absence of VPA.

The PCA of the transcriptomes of day 0 and day 14 based on all microarray data showed significant differences between undifferentiated hESCs and hiPSCs (day 0) and their differentiated (14 days) cells. Interestingly, differences in the transcriptomes were fairly uniform for all three cell lines. The differentially expressed genes on day 14, designated as developmental genes, showed significant differences between the hESCs and both hiPSC lines, although the transcriptomes in undifferentiated hESCs, and both hiPSCs lines were very similar. A GO analysis of the upregulated genes at day 14 encompassed more than 30% of embryonic development-related biological processes (developmental GOs) in all three cell lines. Further analysis of these developmental genes in hESCs and hiPSCs revealed that approximately 50% of these similarities were attributed to upregulated developmental GOs, irrespective of whether hESCs or hiPSCs had been differentiated. Similarities were also observed for KEGG pathways for all three cell lines. Several common GOs, such as “anatomical system development”, “nervous system development”, “embryonic morphogenesis”, related to embryonic development, were identified in the developmental genes.

A CellNet analysis revealed almost uniform ESC scores for all three undifferentiated cell lines on day 0, and a uniform reduction of ESC scores on day 14 of differentiation, along with an increase in cell/tissue type scores, such as fibroblast, lung, skin, kidney, heart and liver. A few developmental genes from all three cell lines were from the ectoderm; mesoderm and endoderm lineages, indicating a partial recapitulation of in vivo embryonic development at the transcriptomic level. Our CellNet analysis showed that hiPSCs and hESCs have similar differentiation potential, suggesting that hiPSCs can recapitulate developmental processes of differentiated hESCs.

VPA has teratogenic potential, inducing spina bifida at steady state concentrations of 0.51 ± 0.17 mM in humans. In this study, we used a C_max_ approximately two times above this level [26, 27]. Exposure to VPA during the 14-day differentiation period resulted in deregulation of developmental genes, with opposing induction, i.e., upregulated developmental genes were downregulated, while downregulated developmental genes were upregulated. Very few VPA deregulated genes were common to all three cell lines. We found that more downregulated genes belonged to embryonic development-related GOs than upregulated ones. This clearly shows the inhibitory effects of VPA on differentiation. The common VPA upregulated developmental genes in all three cell lines were associated with anatomical structure and nervous system development, whereas VPA downregulated developmental genes were related to nervous system development, neurogenesis, and brain development. These results are consistent with our earlier published findings, demonstrating that VPA repressed neural tube and dorsal forebrain developmental genes, and upregulated axonogenesis and ventral forebrain associated genes in differentiating hESCs [13].

However, we also noted differences between the three cell lines in genes associated with embryonic development and regulated by VPA. Specifically, upregulated genes associated with neural crest cell development were identified in differentiated H9 ESCs, whereas oligodendrocyte differentiation and germ cell development were identified in differentiated IMR90 and foreskin hiPSCs, respectively. Downregulated genes associated with telencephalon development were identified in differentiated H9 hESCs, whereas genes involved in the metencephalon development and heart tube development were identified in IMR90 and foreskin hiPSCs, respectively. Clearly, GOs identified in hESCs and hiPSCs do not allow a quantification of the toxic effect of developmental toxicants.

Given that a conclusion as to whether hiPSCs can replace hESCs for developmental toxicity testing based on a GO analysis is not possible, we proposed the use of two indices: *D*_*p*_ and *D*_*i*_ based on VPA deregulated developmental genes. *D*_*p*_ represents the intersection of VPA-deregulated genes with developmental genes and its value directly correlates with the developmental toxicity potential. *D*_*i*_ represents the ratio of developmental genes among VPA deregulated total genes; a high overrepresentation value means that VPA preferentially deregulates developmental genes. *D*_*p*_ and *D*_*i*_ values were estimated for various fold change values for the developmental genes (> 2, > 5 and > 10). *D*_*p*_ values showed a linear increase for the same cell line with increasing fold change, but varied among cell lines. Interestingly, the *D*_*i*_ values were similar for all three cell lines, for any given fold change value. The greatest increase in *D*_*i*_ values occurred for a fold change from two to five. There also was a moderate increase in *D*_*i*_ from fivefold to tenfold change in developmental genes, indicating that a fivefold change for developmental genes is most critical for the *D*_*i*_ calculation. Thus, this index has strong potential for prediction of developmental toxicants.

Among the VPA-deregulated genes common to all three cell lines, several developmental genes were of particular interest for assessing in vivo observed teratogenic effects of VPA. In particular, we identified two upregulated developmental genes, which become downregulated by VPA (*DOK6* and *BCL2*), and two downregulated developmental genes that become upregulated by VPA *(CLDN10* and *PRKCB*).

Treatment with VPA during pregnancy in women has resulted in teratogenic malformations in newborns, including neural tube defects, microcephaly, ventricular septal defects, craniofacial abnormalities, ear abnormalities and urogenital abnormalities [28]. The gene Docking Protein 6 (*DOK6*), a member of the DOK family, plays a role in Ret tyrosine kinase signalling, which promotes neurite outgrowth (Crowder et al., 2004). In a mouse model, knockdown of *Dok6* by specific RNAi resulted in decreased neurite outgrowth (Li et al., 2010). B-cell CLL lymphoma 2 (*BCL2*) has been described as a key regulator of embryonic development. Even though *Bcl2* knockout in mice is not lethal, it still exhibits various malformations during postnatal development, including growth retardation, smaller ears, atrophic thymus and spleen [29, 30]. *Bcl2* knockout mice exhibited progressive degeneration of motor neurons of the facial region [31]. Claudin 10 (*CLDN10)* is a downregulated developmental gene that becomes upregulated by VPA. Gain of function studies in chicken demonstrate that *CLDN10* is crucial for normal heart tube looping [32]. The Protein Kinase C Beta (*PRKCB*) is also upregulated by VPA, and recently, significant copy number variation has been found in human patients with ventricular septal defects [33]. In accordance with our findings, it has been established that VPA stimulates *PRKCB* in several cell types [34, 35].

## Conclusions

Our results suggest that even though hESCs and hiPSCs show common and distinct differentiation transcriptomic profiles, the developmental hazard of the test compounds can be determined by estimating *D*_*i*,_ irrespective of whether hESCs or hiPSCs are used in the test system. Both *D*_*p*_ and *D*_*i*_ provide a novel approach to quantify the potential of drugs to cause developmental hazards based on pluripotent stem cells and transcriptomics. In addition, we show that key developmental genes deregulated by VPA may be potential players in the phenotypic malformations observed after patient treatment with VPA.

## Additional files

Additional file 1: Figure S1. Tissue classification based on CellNet analysis for human embryonic stem cells (hESCs) and human induced pluripotent stem cells (hiPSCs). Analysis was performed using the.CEL files of undifferentiated H9 ESCs, foreskin hiPSCs and IMR90 hiPSCs (day 0), as well as the differentiated cells (day 14). Although the tissue classification scores were < 0.2, hESCs and hiPSCs revealed an increase in score during differentiation (day 14), compared with day 0. Higher tissue classification scores for neuron, fibroblast, lung, skin and heart tissue were found in the IMR90 hiPSCs, compared to foreskin hiPSCs and H9 ESCs. **Figure S2.** H9 hESCs, IMR90 and foreskin hiPSCs were differentiated for 14 days, exposed to valproic acid (VPA) during differentiation. Samples collected on day 0 and day 14, as indicated in Fig. 1a, were used for whole transcriptome analysis. The data structure of all transcriptome data sets was dimensionally reduced and presented as a two-dimensional principle component analysis (2D-PCA) diagram. The PCA illustrates a relatively large distance between hESCs and hiPSCs on day 0, indicating initial differences in transcriptome profile, however 14 days of differentiation resulted in a large distance between day 0 and day 14 in all cell lines, related to changes along the PC1 axis. **Figure S3.** Overlap analysis of “developmental” probe sets (D-PS) in H9 hESCs, IMR90 and foreskin hiPSCs (for absolute fold change ≥5, *p* < 0.05) deregulated by VPA. D-PS were identified, as described in Additional file 2: Table S2 and VPA-affected genes (T-genes) were identified, as described in Additional file 2: Table S3. The overlap of upregulated T-genes with up- (*red*) and down- (*blue*) regulated D-PS, as well as the overlap of downregulated T-genes with up- and downregulated D-PS was calculated for all three cell lines. The data are expressed as the fraction of D-PS affected by VPA. (PPTX 271 kb)
Additional file 2: Table S1. “Developmental probe sets” significantly deregulated on day 14 with respect to day 0 for foreskin, IMR90 and H9 cell lines (absolute fold change ≥ 2, false discovery rate-corrected *p* value < 0.05). **Table S2.** “Deregulated probe sets” by valproic acid (VPA) on day 14 with respect to control on day 14 in foreskin, IMR90 and H9 cell lines (absolute fold change ≥ 2, false discovery rate-corrected *p* value < 0.05). **Table S3.** List of significant biological processes (Gene Ontology categories; GOs) captured by “upregulated developmental genes” in foreskin, IMR90 and H9 cell lines (absolute fold change ≥ 2, false discovery rate-corrected *p* value < 0.05). **Table S4.** List of significant biological processes (Gene Ontology categories; GOs) captured by “downregulated developmental genes” in foreskin, IMR90 and H9 cell lines (absolute fold change ≥ 2, false discovery rate-corrected *p* value < 0.05). **Table S5.** Differentially expressed developmental genes belonging to the three germ layers and deregulated by valproic acid (VPA) (absolute fold change ≥ 2, false discovery rate-corrected *p* value < 0.05). **Table S6.** Probe sets significantly deregulated on day 0 among foreskin, IMR90 and H9 cell lines (absolute fold change ≥ 2, false discovery rate-corrected *p* value < 0.05). **Table S7.** List of significant biological processes (Gene Ontology categories; GOs) captured by valproic acid (VPA) downregulated probe sets on day 14 in differentiated foreskin, IMR90 and H9 cell lines (absolute fold change ≥ 2, false discovery rate-corrected *p* value < 0.05). **Table S8.** List of significant biological processes (GOs) captured by valproic acid (VPA) downregulated probe sets on day 14 in differentiated foreskin, IMR90 and H9 cell lines (absolute fold change ≥ 2, false discovery rate-corrected *p* value < 0.05). (XLSX 1225 kb)

## Abbreviations

BPs: biological processes
DAVID: Database for Annotation, Visualization and Integrated Discovery
DMEM: Dulbecco’s modified Eagle’s medium
FDR: false discovery rate
GOs: gene ontology categories
hESCs: human embryonic stem cells
hiPSCs: human induced pluripotent stem cells
KEGG: Kyoto Encyclopedia of Genes and Genomes
PCA: principal component analysis
PGS: Partek Genomics Suite
VPA: valproic acid

## References

[1] Tafuri G, Trotta F, Leufkens HG (2013). Disclosure of grounds of European withdrawn and refused applications: a step forward on regulatory transparency. Br J Clin Pharmacol.

[2] Hengstler JG, Foth H, Kahl R (2006). The REACH concept and its impact on toxicological sciences. Toxicology.

[3] Leist M, Ringwald A, Kolde R (2013). Test systems of developmental toxicity: state-of-the art and future perspectives. Arch Toxicol.

[4] Adler S, Basketter D, Creton S (2011). Alternative (non-animal) methods for cosmetics testing: current status and future prospects-2010. Arch Toxicol.

[5] Hartung T, Daston G (2009). Are in vitro tests suitable for regulatory use?. Toxicol Sci.

[6] Shukla SJ, Huang R, Austin CP (2010). The future of toxicity testing: a focus on in vitro methods using a quantitative high-throughput screening platform. Drug Discov Today.

[7] Brown ES, Jacobs A, Fitzpatrick S (2012). Reproductive and developmental toxicity testing: from in vivo to in vitro. Altex.

[8] Anson BD, Kolaja KL, Kamp TJ (2011). Opportunities for use of human iPS cells in predictive toxicology. Clin Pharmacol Ther.

[9] Gunaseeli I, Doss MX, Antzelevitch C (2010). Induced pluripotent stem cells as a model for accelerated patient- and disease-specific drug discovery. Curr Med Chem.

[10] Shinde V, Hoelting L, Srinivasan SP et al. Definition of transcriptome-based indices for quantitative characterization of chemically disturbed stem cell development: introduction of the STOP-Toxukn and STOP-Toxukk tests. Arch Toxicol 2016; doi. 10. 1007/s00204-016-1741-8. 10.1007/s00204-016-1741-8 PMC5306084 27188386

[11] Krug AK, Kolde R, Gaspar JA (2013). Human embryonic stem cell-derived test systems for developmental neurotoxicity: a transcriptomics approach. Arch Toxicol.

[12] Balmer NV, Klima S, Rempel E (2014). From transient transcriptome responses to disturbed neurodevelopment: role of histone acetylation and methylation as epigenetic switch between reversible and irreversible drug effects. Arch Toxicol.

[13] Meganathan K, Jagtap S, Srinivasan SP (2015). Neuronal developmental gene and miRNA signatures induced by histone deacetylase inhibitors in human embryonic stem cells. Cell Death Dis.

[14] Meganathan K, Jagtap S, Wagh V (2012). Identification of thalidomide-specific transcriptomics and proteomics signatures during differentiation of human embryonic stem cells. PLoS One.

[15] Shinde V, Klima S, Sureshkumar PS (2015). Human pluripotent stem cell based developmental toxicity assays for chemical safety screening and systems biology data generation. J Vis Exp.

[16] Leist M, Bremer S, Brundin P (2008). The biological and ethical basis of the use of human embryonic stem cells for in vitro test systems or cell therapy. Altex.

[17] Takahashi K, Yamanaka S (2006). Induction of pluripotent stem cells from mouse embryonic and adult fibroblast cultures by defined factors. Cell.

[18] Hogberg HT, Bressler J, Christian KM (2013). Toward a 3D model of human brain development for studying gene/environment interactions. Stem Cell Res Ther.

[19] Schmidt BZ, Lehmann M, Gutbier S, et al. In vitro acute and developmental neurotoxicity screening: an overview of cellular platforms and high-throughput technical possibilities. Arch Toxicol. 2016. doi:10.1007/s00204-016-1805-9. 10.1007/s00204-016-1805-9 27492622

[20] Bilic J, Izpisua Belmonte JC (2012). Concise review: Induced pluripotent stem cells versus embryonic stem cells: close enough or yet too far apart?. Stem Cells.

[21] Shinde V, Stober R, Nemade H (2015). Transcriptomics of hepatocytes treated with toxicants for investigating molecular mechanisms underlying hepatotoxicity. Methods Mol Biol.

[22] da Huang W, Sherman BT, Lempicki RA (2009). Systematic and integrative analysis of large gene lists using DAVID bioinformatics resources. Nat Protoc.

[23] da Huang W, Sherman BT, Lempicki RA (2009). Bioinformatics enrichment tools: paths toward the comprehensive functional analysis of large gene lists. Nucleic Acids Res.

[24] Cahan P, Li H, Morris SA (2014). Cell Net: network biology applied to stem cell engineering. Cell.

[25] Rempel E, Hoelting L, Waldmann T (2015). A transcriptome-based classifier to identify developmental toxicants by stem cell testing: design, validation and optimization for histone deacetylase inhibitors. Arch Toxicol.

[26] Omtzigt JG, Los FJ, Grobbee DE (1992). The risk of spina bifida aperta after first-trimester exposure to valproate in a prenatal cohort. Neurology.

[27] Mawer G, Clayton-Smith J, Coyle H (2002). Outcome of pregnancy in women attending an outpatient epilepsy clinic: adverse features associated with higher doses of sodium valproate. Seizure.

[28] Jentink J, Loane MA, Dolk H (2010). Valproic acid monotherapy in pregnancy and major congenital malformations. N Engl J Med.

[29] Kamada S, Shimono A, Shinto Y (1995). bcl-2 deficiency in mice leads to pleiotropic abnormalities: accelerated lymphoid cell death in thymus and spleen, polycystic kidney, hair hypopigmentation, and distorted small intestine. Cancer Res.

[30] Veis DJ, Sorenson CM, Shutter JR (1993). Bcl-2-deficient mice demonstrate fulminant lymphoid apoptosis, polycystic kidneys, and hypopigmented hair. Cell.

[31] Michaelidis TM, Sendtner M, Cooper JD (1996). Inactivation of bcl-2 results in progressive degeneration of motoneurons, sympathetic and sensory neurons during early postnatal development. Neuron.

[32] Collins MM, Baumholtz AI, Simard A (2015). Claudin-10 is required for relay of left-right patterning cues from Hensen's node to the lateral plate mesoderm. Dev Biol.

[33] An Y, Duan W, Huang G (2016). Genome-wide copy number variant analysis for congenital ventricular septal defects in Chinese Han population. BMC Med Genomics.

[34] Larsson P, Ulfhammer E, Magnusson M (2012). Role of histone acetylation in the stimulatory effect of valproic acid on vascular endothelial tissue-type plasminogen activator expression. PLoS One.

[35] Monti B, Polazzi E, Contestabile A (2009). Biochemical, molecular and epigenetic mechanisms of valproic acid neuroprotection. Curr Mol Pharmacol.

