# Supplementary material for: Comparison of a teratogenic transcriptome-based predictive test based on human embryonic versus inducible pluripotent stem cells
Source: Stem Cell Res Ther. 2016 Dec 30;7:190. doi: 10.1186/s13287-016-0449-2 (PMC5203708; doi:10.1186/s13287-016-0449-2)
Supplement: Additional file 1: Figure S1. — Tissue classification based on CellNet analysis for human embryonic stem cells (hESCs) and human induced pluripotent stem cells (hiPSCs). Analysis was performed using the.CEL files of undifferentiated H9 ESCs, foreskin hiPSCs and IMR90 hiPSCs (day 0), as well as the differentiated cells (day 14). Although the tissue classification scores were < 0.2, hESCs and hiPSCs revealed an increase in score during differentiation (day 14), compared with day 0. Higher tissue classification scores for neuron, fibroblast, lung, skin and heart tissue were found in the IMR90 hiPSCs, compared to foreskin hiPSCs and H9 ESCs. Figure S2. H9 hESCs, IMR90 and foreskin hiPSCs were differentiated for 14 days, exposed to valproic acid (VPA) during differentiation. Samples collected on day 0 and day 14, as indicated in Fig. 1a, were used for whole transcriptome analysis. The data structure of all transcriptome data sets was dimensionally reduced and presented as a two-dimensional principle component analysis (2D-PCA) diagram. The PCA illustrates a relatively large distance between hESCs and hiPSCs on day 0, indicating initial differences in transcriptome profile, however 14 days of differentiation resulted in a large distance between day 0 and day 14 in all cell lines, related to changes along the PC1 axis. Figure S3. Overlap analysis of “developmental” probe sets (D-PS) in H9 hESCs, IMR90 and foreskin hiPSCs (for absolute fold change ≥5, p < 0.05) deregulated by VPA. D-PS were identified, as described in Additional file 2: Table S2 and VPA-affected genes (T-genes) were identified, as described in Additional file 2: Table S3. The overlap of upregulated T-genes with up- (red) and down- (blue) regulated D-PS, as well as the overlap of downregulated T-genes with up- and downregulated D-PS was calculated for all three cell lines. The data are expressed as the fraction of D-PS affected by VPA. (PPTX 271 kb) [file 13287_2016_449_MOESM1_ESM.pptx]

## Slide 1
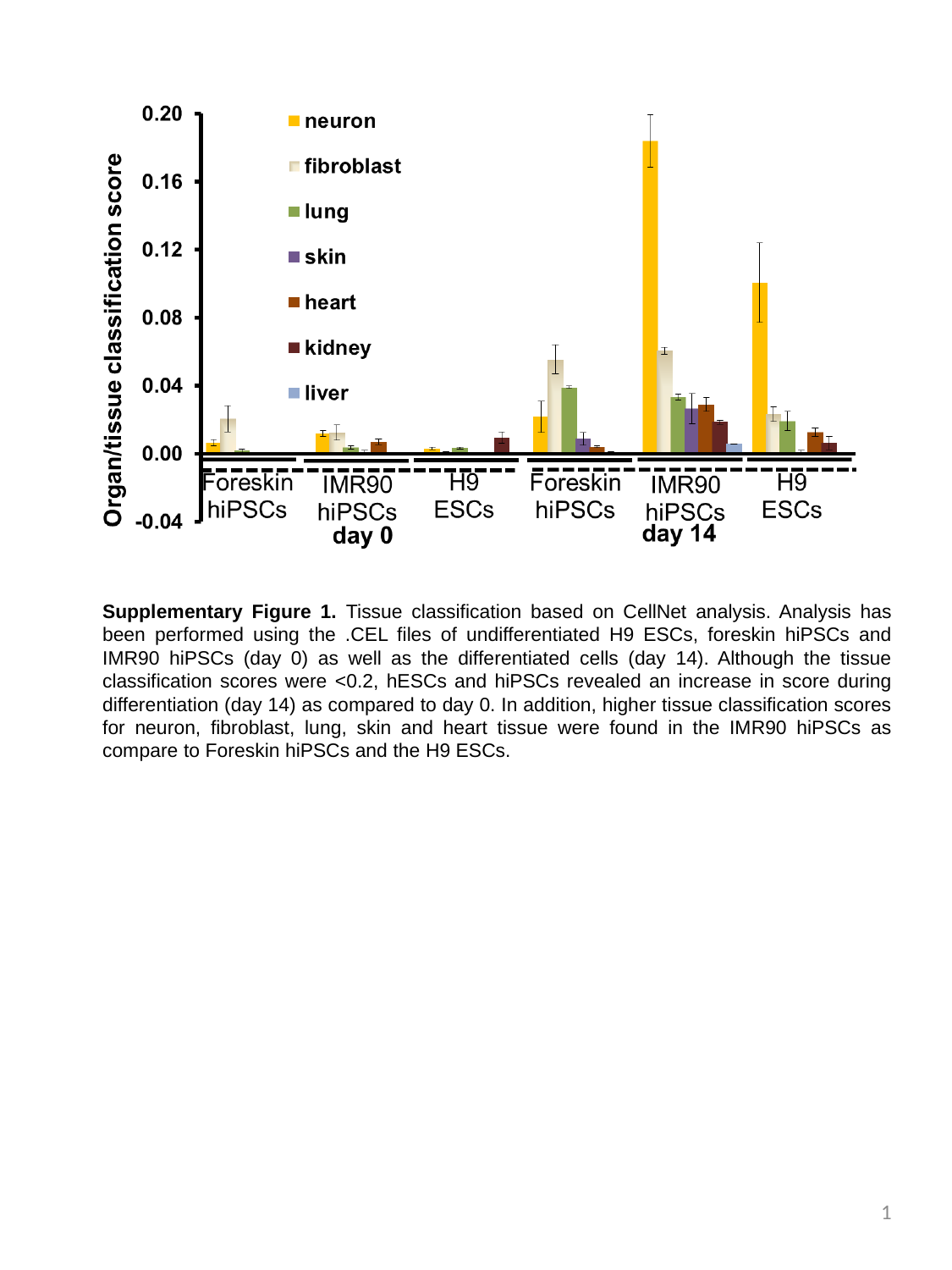

Supplementary Figure 1. Tissue classification based on CellNet analysis. Analysis has been performed using the .CEL files of undifferentiated H9 ESCs, foreskin hiPSCs and IMR90 hiPSCs (day 0) as well as the differentiated cells (day 14). Although the tissue classification scores were <0.2, hESCs and hiPSCs revealed an increase in score during differentiation (day 14) as compared to day 0. In addition, higher tissue classification scores for neuron, fibroblast, lung, skin and heart tissue were found in the IMR90 hiPSCs as compare to Foreskin hiPSCs and the H9 ESCs.
1

## Slide 2
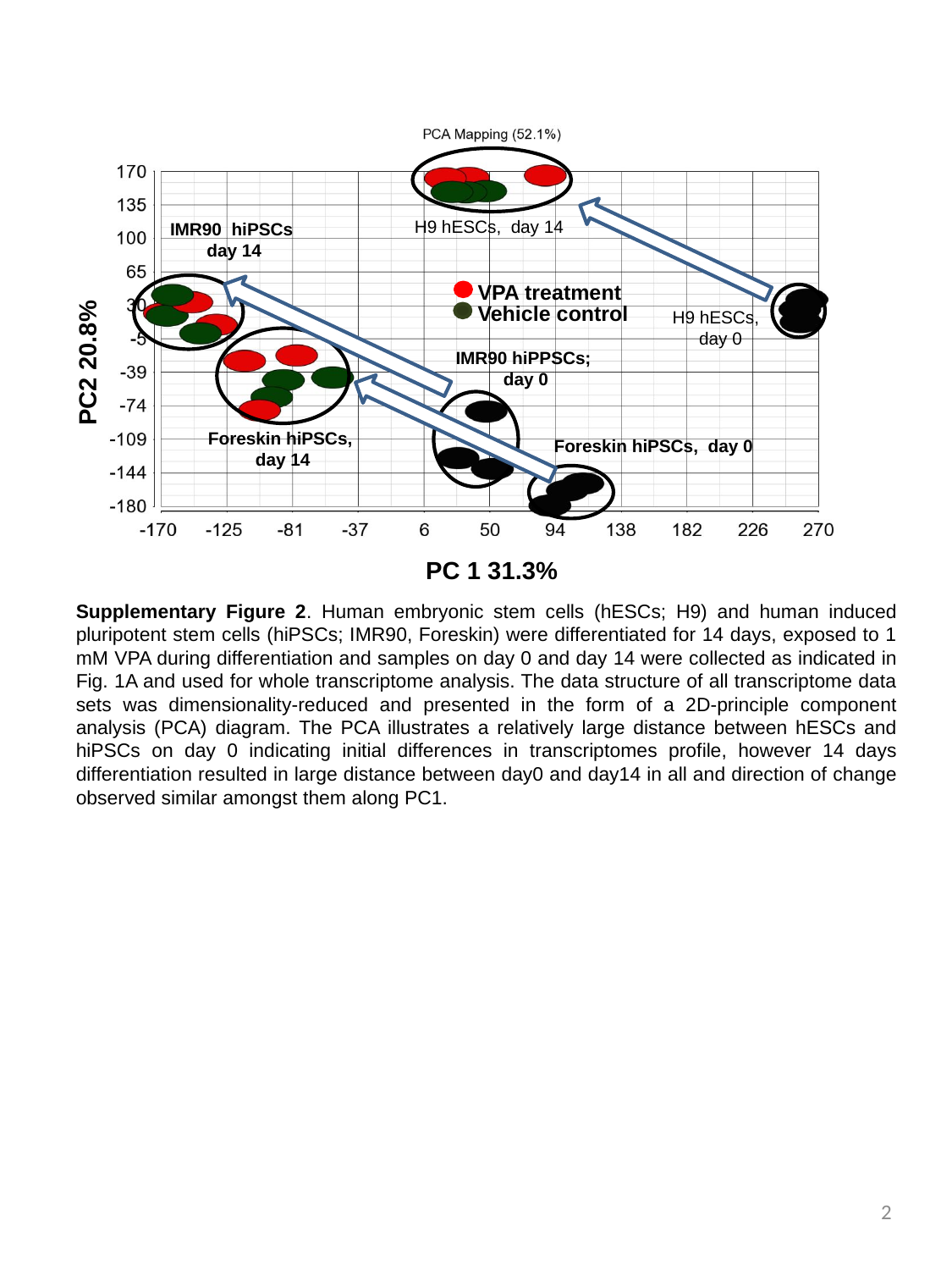

PC2 20.8%
PC 1 31.3%
H9 hESCs, day 14
IMR90 hiPSCs day 14
H9 hESCs,
day 0
IMR90 hiPPSCs;
day 0
Foreskin hiPSCs, day 14
Foreskin hiPSCs, day 0
VPA treatment
Vehicle control
Supplementary Figure 2. Human embryonic stem cells (hESCs; H9) and human induced pluripotent stem cells (hiPSCs; IMR90, Foreskin) were differentiated for 14 days, exposed to 1 mM VPA during differentiation and samples on day 0 and day 14 were collected as indicated in Fig. 1A and used for whole transcriptome analysis. The data structure of all transcriptome data sets was dimensionality-reduced and presented in the form of a 2D-principle component analysis (PCA) diagram. The PCA illustrates a relatively large distance between hESCs and hiPSCs on day 0 indicating initial differences in transcriptomes profile, however 14 days differentiation resulted in large distance between day0 and day14 in all and direction of change observed similar amongst them along PC1.
2

## Slide 3
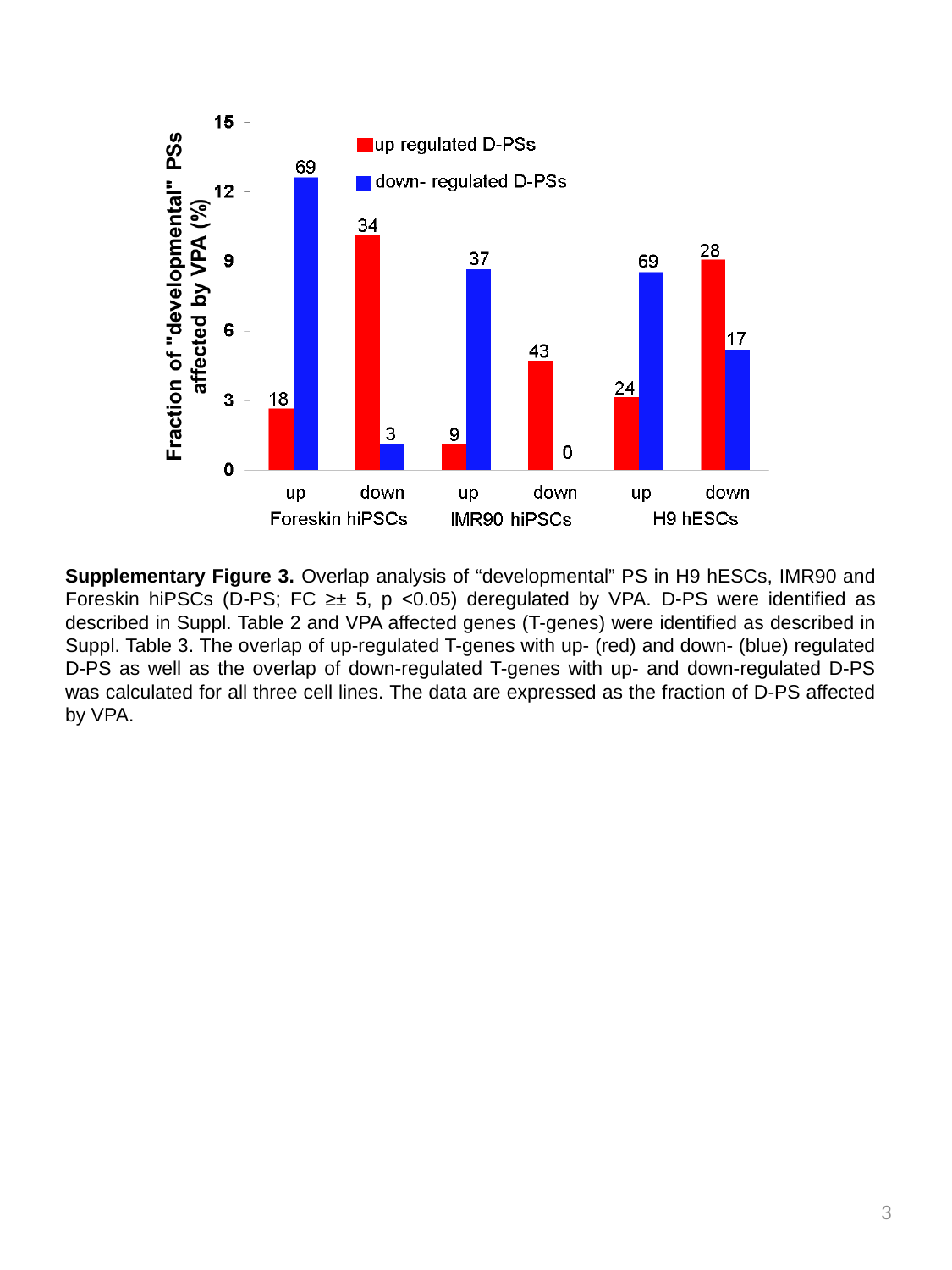

Supplementary Figure 3. Overlap analysis of “developmental” PS in H9 hESCs, IMR90 and Foreskin hiPSCs (D-PS; FC ≥± 5, p <0.05) deregulated by VPA. D-PS were identified as described in Suppl. Table 2 and VPA affected genes (T-genes) were identified as described in Suppl. Table 3. The overlap of up-regulated T-genes with up- (red) and down- (blue) regulated D-PS as well as the overlap of down-regulated T-genes with up- and down-regulated D-PS was calculated for all three cell lines. The data are expressed as the fraction of D-PS affected by VPA.
3
